# Owner points of view and perceived quality of life of diabetic cats pre- and post-hypophysectomy for hypersomatotropism

**DOI:** 10.1093/jvimsj/aalaf006

**Published:** 2026-01-21

**Authors:** Edward Shelton, Rosanne Jepson, David Church, Joe Fenn, Christopher Scudder

**Affiliations:** Department of Clinical Science and Services, Royal Veterinary College, Hatfield AL9 7TA, United Kingdom; Department of Clinical Science and Services, Royal Veterinary College, Hatfield AL9 7TA, United Kingdom; Department of Clinical Science and Services, Royal Veterinary College, Hatfield AL9 7TA, United Kingdom; Department of Clinical Science and Services, Royal Veterinary College, Hatfield AL9 7TA, United Kingdom; Department of Clinical Science and Services, Royal Veterinary College, Hatfield AL9 7TA, United Kingdom

**Keywords:** feline, acromegaly, pituitary, transsphenoidal, diabetic remission

## Abstract

**Background:**

Hypophysectomy provides the most favorable long-term outcome for cats with hypersomatotropism (HST) and concurrent diabetes mellitus (DM).

**Hypothesis/Objectives:**

Assess owner perceptions of quality of life (QoL) of their diabetic cats pre- and post-hypophysectomy for HST.

**Animals:**

Client-owned cats (27 retrospectively, 13 prospectively).

**Methods:**

Owners whose cats had undergone hypophysectomy between 2012 and 2022 for the management of HST with DM were identified. Owner telephone interviews were performed to formulate questions addressing points of view regarding HST and hypophysectomy. The DIAQoL-Pet questionnaire was adjusted to include newly formulated questions and distributed to owners. The questionnaire also was distributed to owners of cats with HST with DM pre-hypophysectomy (T0) and at least 3 months post-hypophysectomy (T1) between March 2023 and November 2024. Item-weighted impact score (IWIS) and an average-weighted impact score (AWIS) were calculated. The AWIS and IWIS at T0 and T1 for prospectively recruited cases were compared using the Wilcoxon signed rank test.

**Results:**

Nearly 92% (22 out of 24) of retrospective group respondents and 100% (10 out of 10) of prospective group respondents would definitely request hypophysectomy again. For paired prospective group responses, IWIS were significantly less negative postoperatively for “worry” (*P* = .02), “pet unwell” (*P* = .03), “worry hypoglycemia” (*P* = .01), and “worry vision” (*P* = .04). The median AWIS before (−2.16; IQR, −4.23 to −1.17) and after (−1.27; IQR, −2.06 to −0.39) hypophysectomy were significantly less negative (*P* = .02).

**Conclusions and clinical importance:**

Most owners perceive hypophysectomy to be a beneficial intervention for their QoL and that of their cat.

## Introduction

Hypersomatotropism (HST) is caused by chronic excessive growth hormone (GH), typically caused by a pituitary somatotroph adenoma or hyperplasia in cats.^1^^,^^2^ The prevalence of HST in cats with diabetes mellitus (DM) has been reported to be between 15% and 25%.^1^^,^^3^^,^^4^ Transsphenoidal hypophysectomy is the treatment option that provides curative potential for cats with HST, with diabetic remission achieved in 71%-91% of cats.^5^^,^^6^ Median survival times (MST) of 853 and 1347 days have been reported after hypophysectomy with mortality rates in the 4 weeks postoperatively between 4% and 15%.^5^^,^^6^ Cats post-hypophysectomy require lifelong glucocorticoid and thyroid hormone supplementation. Long-term desmopressin supplementation has been reported in 58%-72% of cats post-hypophysectomy, with the remainder being able to be weaned off this medication.^5^^,^^6^

Quality of life (QoL) in humans after pituitary adenomectomy for the treatment of acromegaly has been shown to remain lower than for the general population, with psychosocial well-being (relating to appearance and physical function) negatively impacting QoL.^7^^,^^8^ Although no HST specific QoL tool has been validated in veterinary medicine, the DIAQoL Pet questionnaire represents a validated measure of owner perceived QoL of themselves and their diabetic cats.^9^ Quality of life was reported to not improve after treatment with cabergoline in a small cohort of cats with HST, whereas hypophysectomy has been reported to improve QoL of cats compared with medical management using insulin or cabergoline.^10^^,^^11^ The study specifically evaluating QoL in cats with HST only incorporated retrospective QoL assessment, with 14 out of 127 cats that had undergone hypophysectomy being described.^11^ Because hypophysectomy presents substantial lifestyle and financial implications for owners because of ongoing postoperative monitoring and medication requirements, it is important to understand whether owners of cats that have undergone this procedure perceive the intervention to have been beneficial or not.

Our aim was to evaluate owners’ perceptions of QoL of their diabetic cats pre- and post-hypophysectomy for HST using a modified version of the DIAQoL-Pet questionnaire. We hypothesized that owners would perceive that hypophysectomy was beneficial to the QoL of their cats.

## Materials and methods

### Retrospective and prospective cohorts

Ours was a dual retrospective cross-sectional observational and prospective longitudinal observational study. Owners of cats undergoing hypophysectomy for HST and DM at the Queen Mother Hospital for Animals, Royal Veterinary College between April 2, 2012 and December 7, 2022 were identified by a review of hospital records. Cases were excluded if the cat was not diabetic at the time of hypophysectomy and if hypophysectomy was performed for a reason other than HST. Cats known to have died >6 months before the time of questionnaire distribution were not included in an attempt to minimize recall bias and to avoid distressing owners by asking them to complete the questionnaire.

A prospective cohort of owners of cats with HST and DM presented to the Queen Mother Hospital for Animals, Royal Veterinary College for hypophysectomy surgery between March 15, 2023 and November 5, 2024 also were enrolled into the study.

Ethical approval for this study was obtained from the Royal Veterinary College Social Sciences Research Ethical Review Board (unique reference number: SR2023-0036).

### Questionnaire design and distribution to retrospective cohort

The DIAQoL-pet questionnaire was used as the template for the HST and DM QoL questionnaire.^9^ Questions containing reference to insulin administration were amended so that all medications being administered were considered by owners. Two overview questions relating to general and condition dependent QoL were retained. Twenty-eight of the original 29 DIAQoL-pet items (specific DM QoL related issues) were retained. A free-text question “Is there anything else you would like to say about your experiences of life with a pet with diabetes / experiences of life with a pet who has undergone hypophysectomy?” was included at the close of the questionnaire.

Scripted telephone interviews then were performed with randomly selected owners of eligible cats within the retrospective cohort to screen for additional owner points of view around hypophysectomy in their cats and allow formulation of questions to cover areas not included within the amended DIAQoL-Pet Questionnaire. Telephone interviews were ceased once subjective data saturation was reached, defined as the point at which new themes of interest were no longer emerging.^12^ Additional questions relating to desmopressin administration, hunger, and comorbidities were formulated after discussion among the authors based on themes raised in telephone interviews (with “hunger” being the original DIAQoL-pet item not included on initial questionnaire adjustment). Questions to clarify alive or dead status of cats were added alongside a question “Given the choice again, would you proceed with hypophysectomy surgery again?”. The structure of the telephone interviews, final questionnaire, and full questionnaire text are included in the Supplementary material.

The resulting electronic questionnaire was distributed by email to all eligible owners in the retrospective cohort, including those participating in telephone interviews, between March and July of 2023.

### Questionnaire distribution to prospective cohort

Owners eligible for inclusion in the prospective cohort were asked to complete the questionnaire before their cat’s discharge from the hospital at the time of hypophysectomy based on recollections of QoL before surgery (T0). Owners of cats enrolled up until the August 20, 2024 were also asked to complete the questionnaire again at a time point at least 3 months post-hypophysectomy (T1).

### Clinical variables

The following clinical variables were recorded when available: sex, breed, body weight at time of hypophysectomy, pituitary height (mm), serum insulin-like growth factor 1 (IGF1) concentration pre-hypophysectomy and at follow up (at least 4 weeks post-hypophysectomy), insulin type and dose (units/kg) at time of hypophysectomy and at the time of post-hypophysectomy questionnaire completion (or at the time of the cat’s death), and whether diabetic remission (normoglycemia without exogenous insulin or oral sodium-glucose cotransporter-2 (SGLT2) inhibitor administration for at least 4 weeks) had occurred. The time from hypophysectomy to questionnaire completion in days was recorded for the retrospective cohort and the postoperative questionnaire responses (T1) in the prospective cohort. Pre-operative questionnaire responses (T0) from the prospective cohort were recorded as 0 months from hypophysectomy. For the retrospective cohort, age was recorded as the cat’s age at the time of questionnaire completion or age at time of death if the cat had died or been euthanized before questionnaire completion. For the prospective cohort, the age at the time of T1 was recorded. Concentrations of IGF1 >2000 ng/mL were recorded as 2001 ng/mL and concentrations < 15 ng/mL recorded as 15 ng/mL for the purposes of data analysis. Questionnaire responses were screened to ensure no dual responses for the retrospective cohort. If a dual response was identified, then the owner’s most recent response was included.

### Statistical analysis

Data distribution was assessed using Shapiro–Wilk tests and histograms. Data with a non-normal distribution are presented as median and IQR, and data with a normal distribution as mean and SD. The proportions of responses to questions are presented as percentages. Free-text responses were reviewed and organized into common themes. Item weighted impact scores (IWISs) for each of the 28 items utilizing a Likert scale were calculated by multiplying frequency and importance ratings. The possible IWIS range for 25 negatively weighted items was −12 to 0 and for 3 positively weighted items the possible IWIS range was 0 to 12. The magnitude of an IWIS result indicates the degree of impact of that item on QoL (with results <0 being negatively impactful and results >0 positively impactful). An average weighted impact score (AWIS) for each questionnaire response was calculated by division of all IWISs by the total number of items as previously described.^9^ The IWIS and AWIS pre (T0) and post (T1) hypophysectomy were compared using related samples Wilcoxon signed rank tests. The median of the difference between T0 and T1 IWIS and AWIS values (effect estimate) and 95%CIs also were calculated. Statistical significance was defined as *P* < .05. Statistical analyses were performed using a commercially available statistical analysis software (SPSS, version 30, IBM and GraphPad Prism version 10.2.3).

## Results

### Retrospective population

One-hundred and twenty-one cats were identified. Cats were excluded due to having HST but not DM (*n* = 3), undergoing hypophysectomy for pituitary dependent hyperadrenocorticism (*n* = 3) or being known to have died or been euthanized >6 months after the start of the study (*n* = 71). Of 44 remaining cases, 2 were excluded because inability to contact the owners by email. After 2 telephone interviews, the themes of desmopressin eye drop administration and hunger had been identified as additional owner points of view not included within the amended DIAQoL-Pet questionnaire. Three additional telephone interviews were conducted with no new themes being identified, and at this point it was deemed that data saturation had been reached. Forty-two owners received the QoL questionnaire by email and 29 responses were received. One response was excluded because the cat had died >6 months earlier and 1 dual response was identified and excluded from analysis, leaving 27 responses for analysis.

The median time from hypophysectomy to survey completion was 1409 days (IQR, 821-1933 days; range, 301-2932 days). Two of 27 (7%) owners reported their cats to have died in the preceding 6 months and completed the questionnaire 51 and 149 days, respectively, after their cats died. Twenty four of 27 (89%) cats were male and 3 out of 27 (11%) were female, there were 5 British short hairs, 2 cross breeds, 5 domestic short hairs, 12 domestic long hairs, 2 Maine Coon, and 1 Siamese cat. Additional population characteristics are included in Table 1.

**Table 1:** Clinical characteristics of 27 diabetic cats that underwent hypophysectomy for treatment of hypersomatotropism in retrospective cohort.

**Clinical characteristic**	***N*, %**	**Mean ± SD or median (IQR)**
**Age, years^a^**		13.9 ± 2.1
**Body weight, kg**		5.4 ± 1.2
**Pituitary height, mm**		5.9 (5.0-7.4)
**IGF1 pre-operatively, ng/mL**		1819 (1410-2001)
**IGF1 at follow up, ng/mL**		25 (15-54)
**Insulin type**		
**Lente insulin**	14 (52)	
**Protamine zinc**	12 (44)	
**Glargine U100**	1 (4)	
**Insulin dose (units/kg) pre-operatively**		1.1 (0.7-1.3)
**Insulin dose (units/kg) at follow up**		0.0 (0.0-0.0)
**Achieved diabetic remission**	24 (89)	

^a^The mean age is the age of cats at the time of owner questionnaire completion (or age at time of death in the case of the 2 deceased cats). Data are expressed as mean ± SD in the case of normally distributed data and median (IQR) for non-normally distributed data. Abbreviation: IGF1 = insulin-like growth factor 1.

### Retrospective DIAQOL-pet questionnaire responses

In answer to the question “in general I feel that the quality of my pet’s life is,” of 27 respondents 1 out of 27 (3.7%) answered “fairly good,” 6 out of 27 (22.2%) answered good, and 20 out of 27 (74.1%) answered “as good as it could possibly be.” In answer to the question “if your pet did not have diabetes and/or had hypophysectomy, his/her quality of life would be,” of 27 respondents, 4 out of 27 (14.8%) answered “the same,” 2 out of 27 (7.4%) answered “quite a lot better”, and 21 out of 27 (77.8%) answered “a great deal better.”

The proportion of frequency and importance rating responses and median IWIS values for the 28 QoL items are included in Table 2. The IWIS items found to be most negatively impacting owner perceptions of their cat’s QoL after hypophysectomy were those relating to “friends and family,” “drinking,” “urinate,” and “social life.” The median AWIS was −1.11 (IQR, −1.86 to −0.57).

**Table 2:** Descriptive statistics for retrospective cohort questionnaire responses (*n* = 27) from owners of diabetic cats that underwent hypophysectomy for treatment of hypersomatotropism.

**Item**	**% Frequency**				**% Importance rating**					**Median IWIS (IQR)**
	**All the time**	**Often**	**Occasionally**	**Never/don’t know**	**Not at all important/not applicable**	**Low importance**	**Moderately important**	**Important**	**Very important**	
**Worry**	11.1	25.9	22.2	40.7	29.6	18.5	22.2	11.1	18.5	−1.00 (−4.00 to 0.00)
**No treats**	7.4	11.1	33.3	48.1	37.0	33.3	7.4	14.8	7.4	0.00 (−1.00 to 0.00)
**Conditions restrict life**	18.5	14.8	55.6	11.1	3.7	40.7	22.2	18.5	14.8	−2.00 (−4.00 to −1.00)
**Medication discomfort**	0	0	40.7	59.3	40.7	22.2	7.4	7.4	22.2	0.00 (−1.00 to 0.00)
**Medication worries**	0	3.7	33.3	63.0	29.6	25.9	7.4	7.4	29.6	0.00 (−1.00 to 0.00)
**Resent medications**	0	3.7	18.5	77.8	48.1	25.9	3.7	3.7	18.5	0.00 (0.00 to 0.00)
**Restrict your activities**	22.2	11.1	51.9	14.8	3.7	37.0	25.9	14.8	18.5	−2.00 (−4.00 to −1.00)
**More control**	0	14.8	22.2	63.0	40.7	11.1	14.8	22.2	11.1	0.00 (−2.00 to 0.00)
**Pet’s moods**	0	7.4	51.9	40.7	25.9	14.8	22.2	18.5	18.5	−2.00 (−3.00 to 0.00)
**Pet unwell**	0	7.4	18.5	74.1	59.3	7.4	11.1	11.1	11.1	0.00 (−1.00 to 0.00)
**Boarding kennels**	37.0	0	11.1	51.9	37.0	14.8	3.7	18.5	25.9	0.00 (−9.00 to 0.00)
**Friends and family**	37.0	11.1	18.5	33.3	25.9	18.5	7.4	14.8	33.3	−3.00 (−12.00 to 0.00)
**Hypoglycemia**	0	0	11.1	88.9	59.3	7.4	7.4	3.7	22.2	0.00 (0.00 to 0.00)
**Active day**	7.4	0	0	92.6	92.6	0	0	3.7	3.7	0.00 (0.00 to 0.00)
**Extra things (positive item)**	3.7	18.5	33.3	44.4	37.0	22.2	7.4	14.8	18.5	1.00 (0.00 to 4.00)
**Drinking**	29.6	14.8	33.3	22.2	14.8	22.2	14.8	25.9	22.2	−3.00 (−8.00 to −1.00)
**Urinate**	25.9	11.1	37.0	25.9	18.5	22.2	11.1	25.9	22.2	−3.00 (−6.00 to 0.00)
**Weight loss**	0	7.4	14.8	77.8	48.1	14.8	3.7	7.4	25.9	0.00 (0.00 to 0.00)
**Future care**	0	11.1	22.2	66.7	37.0	7.4	11.1	14.8	29.6	0.00 (−2.00 to 0.00)
**Worry hypo**	3.7	7.4	22.2	66.7	51.9	11.1	7.4	18.5	11.1	0.00 (−3.00 to 0.00)
**Worry DKA**	0	7.4	11.1	81.5	63.0	7.4	11.1	7.4	11.1	0.00 (0.00 to 0.00)
**Worry vision**	0	7.4	25.9	66.7	48.1	18.5	3.7	14.8	14.8	0.00 (−1.00 to 0.00)
**Play less**	0	11.1	3.7	85.2	40.7	14.8	3.7	11.1	29.6	0.00 (0.00 to 0.00)
**Play more (positive item)**	7.4	22.2	25.9	44.4	22.2	25.9	11.1	22.2	18.5	1.00 (0.00 to 4.00)
**Social life**	18.5	22.2	29.6	29.6	29.6	14.8	0	33.3	22.2	−3.00 (−6.00 to 0.00)
**Working life**	3.7	7.4	18.5	70.4	59.3	0	11.1	18.5	11.1	0.00 (−2.00 to 0.00)
**Special Bond (positive item)**	48.1	25.9	14.8	11.1	3.7	7.4	11.1	25.9	51.9	6.00 (4.00 to 12.00)
**Costs**	14.8	11.1	37.0	37.0	33.3	22.2	14.8	11.1	18.5	−1.00 (−6.00 to 0.00)

Abbreviation: IWIS = item-weighted impact score.

### Retrospective additional owner points of view

Of 24 respondents to the question “does your pet currently receive DDAVP/ desmopressin eye drops,” 14 out of 24 (58.3%) answered yes and 10 out of 24 (41.7%) answered no. When asked “do you worry more about administering DDAVP/ desmopressin eye drops than you do when administering insulin/ other medications required after hypophysectomy,” 12.5% of respondents answered “often,” 25.0% of respondents answered “occasionally,” and 62.5% of respondents answered “never.” In answer to the question “compared to before developing acromegaly, do you feel that your pet’s appetite is currently,” 5 out of 24 respondents (20.8%) answered “decreased,” 7 out of 24 (29.2%) answered “normal,” 4 out of 24 (16.7%) answered “increased,” and 8 out of 24 (33.3%) answered “very increased”. In answer to the question “given the choice again, would you proceed with hypophysectomy surgery again,” 22 out of 24 respondents (91.7%) answered “yes,” 2 out of 24 (8.3%) answered “unsure.” No respondents answered “no.”

When asked “Do you worry about any concurrent health issues in your pet aside from diabetes and/or their care after hypophysectomy?,” of 24 respondents, 7 out of 24 (25.9%) answered “no” and 17 out of 14 (63.0%) answered “yes.” All respondents answering “yes” provided additional information to the free-text question regarding concurrent health concerns. Health concerns mentioned included cardiac disease (*n* = 2), gastrointestinal disease (*n* = 2), osteoarthritis and/or mobility concerns (*n* = 4), and weight gain (*n* = 5). Full details of responses are included in the Supplementary material.

All 27 respondents provided an answer to the question “Is there anything else you would like to say about your experiences of life with a pet with diabetes / experiences of life with a pet who has undergone hypophysectomy?”. Four owners commented that, without hypophysectomy, they felt they either would not have their cat, would have euthanized their cat or that hypophysectomy had saved their cat’s life. Two owners felt that hypophysectomy had extended their cat’s life. Two owners reported excessive weight gain in their cat leading to decreased mobility. Three owners referenced difficulty in administering desmopressin eye drops and improvement in ease of administration after switching to PO tablets. One owner reported that the management of the cat was easier because the cat no longer required desmopressin. One of two owners who had answered “unsure” to proceeding with hypophysectomy again commented on the impact on their social life because of administration of desmopressin eye drops and difficulty finding someone to look after the cat. The other owner answering “unsure” did not provide an explanation but commented that the cat had entered diabetic remission. Full details of responses can be found in the Supplementary material.

### Prospective population

Owners of 13 cats with HST and DM undergoing hypophysectomy completed the QoL survey based on pre-operative recollections of their cat’s QoL (T0). Paired postoperative responses (T1, completed at least 3 months postoperatively) were available for 10 cases.

The median time from hypophysectomy to survey completion at T1 was 196.5 days (IQR, 100.75-249.0 days; range, 90-396 days). All cats were alive at T1. Nine of 13 (69%) cats were male and 4 out of 13 (31%) were female, there were 6 domestic long hairs, 3 domestic short hairs, 2 Maine Coons, 1 British short hair, and 1 Russian Blue cat. Additional population characteristics are presented in Table 3.

**Table 3:** Clinical characteristics of 13 diabetic cats that underwent hypophysectomy for treatment of hypersomatotropism in prospective cohort.

**Clinical characteristic**	***N*, %**	**Mean ± SD or median (IQR)**
**Age, years**		11.4 ± 2.2
**Body weight, kg**		4.9 ± 1.0
**Pituitary height, mm**		5.5 (4.7-6.2)
**IGF1 pre-operatively, ng/mL**		2001 (1697-2001)
**IGF1 at follow up, ng/mL**		18.5 (15-108.5)
**Insulin type**		
**Lente insulin**	2 (15)	
**Protamine zinc**	6 (46)	
**Glargine U100**	3 (23)	
**Glargine U300**	1 (8)	
**Velaglifozin rather than insulin pre-operatively**	1 (8)	
**Insulin dose (units/kg) pre-operatively**		1.4 (1.1-1.6)
**Insulin dose (units/kg) at follow up**		0.0 (0.0-0.1)
**Achieved diabetic remission**	9 (69)	

Data are expressed as mean ± SD in the case of normally distributed data and median (IQR) for non-normally distributed data. Abbreviations: IGF1, insulin-like growth factor 1.

### Prospective DIAQOL-pet questionnaire responses

Pre-operatively, in answer to the question “in general I feel that the quality of my pet’s life is”, of 13 respondents, 2 out of 13 (15.4%) answered “poor,” 2 out of 13 (15.4%) answered “fairly good,” 3 out of 13 (23%) answered “good,” and 6 out of 13 (46%) answered “as good as it could possibly be.” In answer to the question “if your pet did not have diabetes and/or had hypophysectomy, his/her quality of life would be,” of 13 respondents, 5 out of 13 (38.5%) answered “quite a lot better” and 8 out of 13 (61.5%) answered “a great deal better.”

Postoperatively, in answer to the question “in general I feel that the quality of my pet’s life is,” 4 out of 10 (40%) answered “good” and 6 six out 10 (60%) answered “as good as it could possibly be.” In answer to the question “if your pet did not have diabetes and/or had hypophysectomy, his/her quality of life would be,” 2 out of 10 respondents (20%) answered “the same,” 2 out of 10 (20%) answered “quite a lot better,”, and 6 out of 10 (60%) answered “a great deal better.”

**Figure 1:**
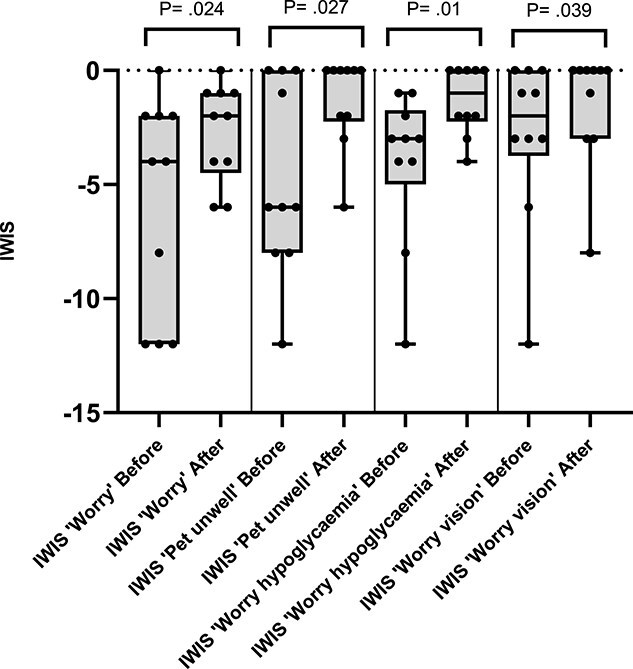
Box and whisker plots for item weighted impact scores (IWIS) where a significant difference was documented on comparison of paired pre- (T0) and post-hypophysectomy (T1) questionnaire responses. Median IWIS identified by solid line and minimum and maximum values by whiskers, individual IWIS by solid dots. IWIS were significantly different postoperatively for “worry” (with 7 positive differences, 1 negative difference, and 2 ties), “pet unwell” (with 6 positive differences, 1 negative difference, and 3 ties), “worry hypoglycemia” (with 9 positive differences and 1 negative difference), and “worry vision” (with 5 positive differences, 0 negative differences, and 5 ties).

**Figure 2:**
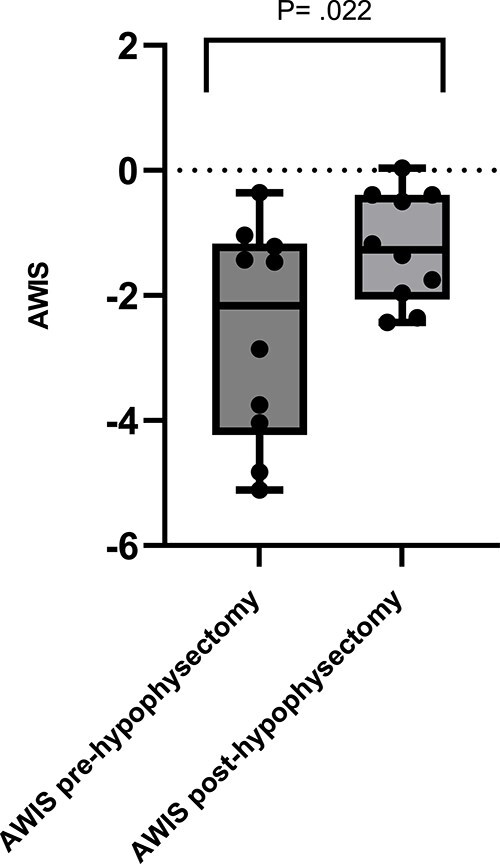
Box and whisker plot showing average weighted impact scores (AWIS) pre- (T0) and post-hypophysectomy (T1) for 10 paired pre- and post-hypophysectomy questionnaire responses. Median AWIS identified by solid line and minimum and maximum values by whiskers, individual AWIS by solid dots. A significant difference in AWIS was documented with 8 positive differences and 2 negative differences.

Median IWIS for paired pre- and post-hypophysectomy questionnaire responses are presented in Table 4. When paired pre and postoperative responses were compared, the IWIS were significantly less negative postoperatively for “worry,” “pet unwell,” “worry hypoglycemia,” and “worry vision” (Figure 1). Median AWIS pre-operatively was −1.46 (IQR, −3.89 to −1.13) when accounting for all 13 responses and −2.16 (IQR, −4.23 to −1.17) when excluding the 3 responses without a paired postoperative response. Median AWIS postoperatively was −1.27 (IQR, −2.06 to −0.39). The AWIS was significantly less negative post-hypophysectomy (*P* = .02), with improvement in AWIS being seen in 8 out of 10 cases (Figure 2). In the 2 cases where a worsening in AWIS was seen, in the first case the AWIS was −0.36 at T0 and −0.39 at T1, and in the second case the AWIS was −1.43 at T0 and −2.43 at T1.

**Table 4:** Prospective cohort median item weighted impact score (IWIS) and average weighted impact score (AWIS) values for 10 available paired pre (T0) and post-hypophysectomy (T1) questionnaire responses from owners of diabetic cats with hypersomatotropism undergoing hypophysectomy.

	**Median (IQR) pre-hypophysectomy**	**Median (IQR) post-hypophysectomy**	**Median of difference between pre- and post-hypophysectomy (effect estimate)**	**95% CI for effect estimate**	***P*-value** ^*^
**IWIS worry**	−4.00 (−12.00 to −2.00)	−2.00 (−4.50 to −1.00)	3.5	0.5-6.0	.02
**IWIS No treats**	−2.00 (−9.00 to −0.75)	−0.50 (−2.25 to 0.00)	2.0	0.0-6.0	.13
**IWIS conditions restrict life**	−2.00 (−6.00 to −0.75)	−2.50 (−4.00 to −0.75)	0.5	−1.0 to 4.5	.29
**IWIS medication discomfort**	−2.00 (−4.5 to 0.0)	−1.00 (−3.25 to −0.75)	1.0	−1.5 to 4.5	.5
**IWIS medication worries**	−2.50 (−4.50 to −0.75)	−2.50 (−3.00 to 0.00)	1.0	−1.0 to 4.5	.23
**IWIS resent medications**	0.00 (−2.25 to 0.00)	0.00 (−0.25 to 0.00)	0.0	−0.5 to 3.0	.46
**IWIS Restrict your activities**	−2.00 (−4.50 to −1.00)	−1.50 (−4.50 to −0.75)	0.5	−1.0 to 2.0	.39
**IWIS More control**	0.00 (−2.50 to 0.00)	0.00 (−1.00 to 0.00)	0.5	0.0-2.0	.1
**IWIS Pet’s moods**	−2.00 (−9.00 to 0.00)	−2.00 (−3.00 to 0.00)	2.0	0.0-5.0	.09
**IWIS Pet unwell**	−6.00 (−8.00 to 0.00)	0.00 (−2.25 to 0.00)	3.0	0.0-6.0	.03
**IWIS Boarding Kennels**	0.00 (0.00 to 0.00)	0.00 (−0.50 to 0.00)	0.0	−1.0 to 0.0	.32
**IWIS Friends and family**	−2.50 (−9.00 to 0.00)	−1.50 (−12.00 to 0.00)	0.0	−6.0 to 5.5	.74
**IWIS Hypoglycemia**	0.00 (0.00 to 0.00)	0.00 (0.00 to 0.00)	0.0	0.0-0.0	1
**IWIS Active day**	0.00 (0.00 to 0.00)	0.00 (0.00 to 0.00)	0.0	0.0-0.0	1
**IWIS Extra things (positive item)**	3.50 (0.75 to 6.50)	3.50 (0.00 to 6.00)	0.0	−3.5 to 3.0	.89
**IWIS Drinking**	−4.50 (−8.25 to 0.00)	−3.00 (−4.50 to −1.75)	0.5	−3.0 to 4.5	.62
**IWIS Urinate**	−4.00 (− 9.00 to 0.00)	−3.00 (−6.00 to −1.50)	1.0	−3.0 to 5.0	.55
**IWIS Weight Loss**	0.00 (−5.25 to 0.00)	0.00 (−3.25 to 0.00)	0.0	−1.0 to 6.0	.47
**IWIS Future care**	−3.00 (−6.00 to 0.00)	0.00 (−1.50 to 0.00)	1.5	−1.5 to 5.5	.25
**IWIS Worry hypo**	−3.00 (−5.00 to −1.75)	−1.00 (−2.25 to 0.00)	2.5	1.0-4.5	.01
**IWIS Worry DKA**	−3.50 (−9.00 to −1.75)	0.00 (−3.00 to 0.00)	3.0	0.0-7.5	.07
**IWIS Worry vision**	−2.00 (−3.75 to 0.00)	0.00 (−3.00 to 0.00)	1.5	0.0-3.0	.04
**IWIS Play less**	0.00 (−5.00 to 0.00)	0.00 (0.00 to 0.00)	0.0	0.0-6.0	.2
**IWIS Play more (positive item)**	0.50 (0.00 to 6.00)	2.00 (0.00 to 5.00)	0.0	−2.0 to 5.0	.83
**IWIS Social life**	−6.00 (−9.00 to 0.00)	−1.50 (−6.00 to −1.00)	2.5	−3.5 to 6.5	.48
**IWIS Working life**	−2.00 (−8.25 to 0.00)	−0.50 (−4.50 to 0.00)	1.0	−1.5 to 4.5	.4
**IWIS Special Bond (positive item)**	0.00 (0.00 to 3.25)	4.50 (0.75 to 9.00)	3.0	−1.0 to 7.5	.12
**IWIS Costs**	−2.50 (−4.50 to 0.00)	−2.00 (4.50 to 0.00)	0.0	−1.0 to 1.5	.78
**AWIS**	−2.16 (−4.23 to 1.17)	−1.27 (−2.06 to −0.39)	1.3	0.3-2.4	.02

^a^ *P*-value for related samples (*n* = 10) Wilcoxon signed rank test.

### Prospective additional owner points of view

In answer to the question “compared to before developing acromegaly, do you feel that your pet’s appetite is currently,” pre-operatively 1 out of 10 respondents (10%) answered “decreased,” 1 out of 10 (10%) answered “normal,” 1 out of 10 (10%) answered “increased,” and 7 out of 10 (70%) answered “very increased.” In answer to the same question postoperatively, 1 out of 10 respondents (10%) answered “decreased,” 5 out of 10 (50%) answered “normal,” 1 out of 10 (10%) answered “increased,” and 3 out of 10 (30%) answered “very increased.”

Postoperatively, all 10 respondents answered “yes” to the question “does your pet currently receive DDAVP/ desmopressin eye drops”. When asked “do you worry more about administering DDAVP/ desmopressin eye drops than you do when administering insulin / other medications required after hypophysectomy, 10% of respondents answered, ‘all the time’, 20% of respondents answered ‘often”, 20% of respondents answered “occasionally” and 50% of respondents answered “never.” In answer to the question “given the choice again, would you proceed with hypophysectomy surgery again”, 10 out of 10 respondents postoperatively answered “yes.”

When asked “Do you worry about any concurrent health issues in your pet aside from diabetes and/or their care after hypophysectomy?”, of 10 respondents pre-operatively, 3 out of 10 (30%) answered “no” and 7 out of 10 (70%) answered “yes.” In answer to the same question postoperatively, 4 out of 10 respondents answered “no” and 6 out of 10 respondents answered “yes.” All owners answering “yes” provided additional details in the free text question regarding concurrent health concerns. Concerns pre-hypophysectomy included cardiac disease (*n* = 3), osteoarthritis and/or mobility concerns including neuropathy (*n* = 3), ocular disease (*n* = 3), and urinary tract disease (*n* = 2). Post-hypophysectomy, weight gain was mentioned as a concern by one owner. Full details of responses are included in the Supplementary material.

Seven owners pre-hypophysectomy and 9 owners post-hypophysectomy provided responses to the question “Is there anything else you would like to say about your experiences of life with a pet with diabetes / experiences of life with a pet who has undergone hypophysectomy?”. Pre-hypophysectomy, 2 owners mentioned excessive hunger, hunger aggression, or both affecting their relationship with their cat. Three owners mentioned difficulty in seeing no response from their cat to insulin injections. Post-hypophysectomy responses were generally positive. Two owners commented on finding medication administration overwhelming initially and concerns raised by individual owners related to postoperative infection, a slower than expected recovery and extreme polyuria and polydipsia with associated challenges in administering desmopressin eye drops. Full details of responses can be found in the Supplementary material.

## Discussion

Ours is the largest study to date reporting QoL in cats after hypophysectomy, and the first to include a prospective cohort. We found that most owners report overall improvement of QoL in their cat after hypophysectomy and that most owners would proceed with hypophysectomy if given the choice again. These findings align with findings of a previous study reporting hypophysectomy may lead to improved QoL when compared to medical management with insulin or cabergoline.^11^

The inclusion of a retrospectively selected group of owners allowed us to analyze the responses of owners who had been living with and managing their cat post-hypophysectomy for a prolonged period of time, because the median time of completion of the questionnaire post-surgery was 1409 days. This cohort of owners will have experienced the acute and chronic consequences of their cat undergoing hypophysectomy. The inclusion of a prospective group of owners allowed for changes in individual IWIS as well as AWIS to be assessed with paired responses pre- and post-hypophysectomy, thereby decreasing the risk of recall bias by allowing the pre-operative responses to act as a control. Postoperative responses were reviewed no earlier than 3 months post-hypophysectomy to allow for owner adjustments to life post-hypophysectomy.

The IWIS most negatively impacting owners perceptions of their cats QoL after hypophysectomy in the retrospective group were not leaving their cats to stay with friends or family, noting increased drinking and urination in comparison to pre-diagnosis and fitting treatments required post-hypophysectomy into their social life. In the original validating study for the DIAQoL-Pet in cats, “social life” was identified as one of the most impactful IWIS, however, “boarding kennel” was the IWIS that most negatively impacted QoL of diabetic cats.^9^ It appears as though similar challenges are faced by owners when managing a cat requiring chronic administration of medication given more than once daily as are faced by those injecting insulin twice daily. Administration of medication is recognized as an area of potential concern to cat owners.^13^ Of the positively weighted IWIS, the item “special bond” achieved the highest score in both the retrospective and prospective groups, although no significant difference was found between pre- and post-hypophysectomy responses in the in prospective cohort. The importance of this item to owners is in agreement with the findings of the original DIAQoL-Pet study.^9^ Although it is notable that it is more a measure of owner satisfaction than cat QoL, it is still an important finding because owner QoL has been shown to impact cat QoL.^14^

Significant improvement of the IWIS for “worry vision” and “worry hypoglycemia” were reported post-hypophysectomy. The concern for decreased vision was unexpected and emphasizes how discrepancies may arise between areas of concern for owners and clinicians. Although lens opacities may occur more commonly in diabetic cats than non-diabetic cats, generally the development of mature cataracts is considered rare in diabetic cats.^15–17^

The improvement of the IWIS “pet unwell” and “worry” aligns with the overall improvement of the AWIS. It appears owners are generally less concerned about managing a cat with hypoadrenocorticism and hypothyroidism, and sometimes also diabetes insipidus, than managing a cat with HST and DM. It is noteworthy that a worsening of AWIS was seen in 2 cases. In 1 case the change in AWIS was marginal and was accompanied by a comment that the owner was delighted with the cat’s progress. In the other case, the change in AWIS was more notable and associated with a more negative post-hypophysectomy IWIS for “worry,” “conditions restrict life,” “pet’s moods,” “pet unwell,” “friends and family,” “drinking,” “urinate,” “worry hypoglycemia,” “play less,” “social life,” “working life,” and “costs.” This owner commented that the cat’s recovery had been slower than expected but the owner was less concerned about the cat’s health and well-being. Both owners answered “yes” to proceeding with hypophysectomy if given the choice again. The variation in AWIS results post-hypophysectomy suggests that hypophysectomy does not, at least immediately, benefit every aspect of owner perceived QoL.

Hunger was not retained as an IWIS within the adapted DIAQoL-Pet questionnaire because of concern for the skewing effect excessive pre-hypophysectomy polyphagia could have on the overall AWIS comparisons pre- and post-hypophysectomy. Polyphagia is recognized in up to 75% of cats with HST and DM and is reported to be extreme in up to 20% of cases, whereas polyphagia of the same severity is not typically recognized in diabetic cats.^1^ Owner telephone interviews emphasized post-hypophysectomy increases in appetite as a potential concern and therefore hunger was assessed using one of the additionally formulated questions. Post-hypophysectomy, appetite was reported to be “increased” compared to before development of acromegaly by 16.7% of owners in the retrospective group and 10% in the prospective postoperative responses, and “very increased” by 33.3% of retrospective and 30.0% of prospective postoperative responses. Furthermore, weight gain was raised as a concern by owners in the retrospective group responses. Hypothalamic damage can result in hyperphagia and weight gain in humans post-surgically after management of pituitary and hypothalamic neoplasia.^18^^,^^19^ Further research is needed to identify the cause of ongoing polyphagia and weight gain in some cats post-hypophysectomy and to identify any treatments that may alleviate these signs given the potential negative impact on QoL.

In our study, discontinuation of desmopressin supplementation was reported in 41.7% of cats in the retrospective cohort, which is similar to the reported proportion of cats requiring desmopressin long-term post-hypophysectomy at another institution.^6^ Desmopressin eye drop administration was a worry for 37.5% of owners in the retrospective group and for 50% of owners in the prospective group postoperatively, and concerns regarding administration also were expressed in the free-text comments. All cats postoperatively in the prospective group and 58.3% of cats in the retrospective group were reported to be receiving desmopressin eye drops. Three owners in the retrospective cohort reported in the free-text comments that their cats were receiving PO desmopressin instead of eye drops. The owners administering desmopressin tablets instead of eye drops commented that they felt this change had been beneficial for them and their cats. Dose efficacy when administering PO desmopressin is considered variable in cats, hence the preference for ocular administration.^20^ It is possible that the proportion of cats remaining on desmopressin treatment post-hypophysectomy is higher than the proportion of cats that have an absolute requirement for desmopressin. Conversely, the ongoing owner worries regarding increased drinking and urination post-hypophysectomy could reflect inadequate control of arginine vasopressin deficiency. Further research is required to better determine which cats have a long-term desmopressin requirement post-hypophysectomy, and if cats do have an ongoing requirement, could more cats be transitioned to PO desmopressin if not tolerant of eye drops.

Our study had several limitations. Question responses were not compulsory, leading to a variable number of available responses for some questions. The questionnaire used has not been validated for use in a population of cats with HST and the findings of initial telephone interviews were not validated with another subgroup of owners. The results obtained may be impacted by owner recall bias and self-selection bias was present because the owners electing to participate in this questionnaire were a highly committed group of owners who wished to improve QoL for their cat and also had shown substantial financial commitment by pursuing hypophysectomy. The potential impact of this factor on our results cannot be quantified. It would have been optimal to have postoperative questionnaire responses completed at the same time points and to assess multiple time points to better assess how owner perceptions of both the QoL of their cat and the commitments associated with post-hypophysectomy management may evolve over time. The absence of a sample size calculation and the small case numbers in the prospective cohort limits how readily our findings can be extrapolated to all owners of cats undergoing hypophysectomy for HST induced DM. For the retrospective cohort, contacting owners of cats known to have died or been euthanized >6 months previously may have provided a higher number of responses. A limitation of this exclusion criterion is the potential exclusion of owners who were less satisfied with their experience of hypophysectomy. Furthermore, an inherent limitation of assessment of QoL in cats is that the results obtained reflect owner perception of QoL rather than the cat’s true QoL. This factor is also a limitation of previous studies utilizing the DIAQoL-Pet or similar tools for assessing QoL in cats.^9^^,^^11^^,^^21^

Our study did not include a control group of diabetic cats with HST not undergoing hypophysectomy. Achieving a sufficient number of cat owners fitting this description was not possible at our institution, although it is possible that the reporting of QoL by owners who did not wish to pursue hypophysectomy may have differed, and subsequently affected the usefulness of this comparison. Almost all cats were receiving insulin with only one cat in the prospective group receiving the SGLT2 inhibitor velaglifozin in place of insulin treatment. Cats with HST induced DM may be expected to have improvement in control of diabetes related clinical signs when treated with an SGLT2 inhibitor, but other clinical signs resulting from excess GH (such as marked polyphagia) may persist despite euglycemia.^22^^,^^23^ Future studies to determine if owner perceptions of QoL would be different pre- and post-hypophysectomy in cats treated with SGLT2 inhibitors rather than insulin would be of interest. Assessing the impact of SGLT2 inhibitor treatment compared to insulin treatment on the QoL of cats with HST for which hypophysectomy is not feasible also would be of interest.

Despite study limitations, attempts at quantitative assessment of QoL in cats undergoing hypophysectomy remains useful to guide owner and clinician decision making. Most owners in our study would proceed again with hypophysectomy for treatment of their cats' HST and DM and perceive it to have improved their cats' QoL. Further research is required to determine how best to manage ongoing polyphagia, weight gain, and desmopressin requirement post-hypophysectomy because these areas were identified potentially to impact QoL negatively post-hypophysectomy.

## Supplementary Material

aalaf006_Supplementary_material_UPDATED

## Off-label antimicrobial declaration

The authors declare no off-label use of antimicrobials.

## Institutional animal care and use committee or other approval declaration

Approval from the Royal Veterinary College Social Sciences Research Ethical Review Board (unique reference number: SR2023-0036).

## References

[1] Niessen SJ, Forcada Y, Mantis P, et al. Studying cat (*Felis catus*) diabetes: beware of the acromegalic imposter. PLoS One. 2015;10:e0127794. 10.1371/journal.pone.0127794 26023776 PMC4449218

[2] Scudder CJ, Mirczuk SM, Richardson KM, et al. Pituitary pathology and gene expression in acromegalic cats. J Endocrin Soc. 2019;3:181-200. 10.1210/js.2018-00226 PMC6316999 30620005

[3] Schaefer S, Kooistra HS, Riond B, et al. Evaluation of insulin-like growth factor-1, total thyroxine, feline pancreas-specific lipase and urinary corticoid-to-creatinine ratio in cats with diabetes mellitus in Switzerland and the Netherlands. J Feline Med Surg. 2017;19:888-896. 10.1177/1098612X16664390 27578200 PMC11104121

[4] Miceli DD, García JD, Rey Amunategui JP, et al. Prevalence of hypersomatotropism and hyperthyroidism in cats with diabetes mellitus from referral centers in Buenos Aires (2020–2022). J Feline Med Surg. 2023;25:1098612X221148565. 10.1177/1098612X221148565 PMC10812081 36779783

[5] Fenn J, Kenny PJ, Scudder CJ, et al. Efficacy of hypophysectomy for the treatment of hypersomatotropism-induced diabetes mellitus in 68 cats. J Vet Intern Med. 2021;35:823-833. 10.1111/jvim.16080 33624865 PMC7995378

[6] van Bokhorst KL, Galac S, Kooistra HS, et al. Evaluation of hypophysectomy for treatment of hypersomatotropism in 25 cats. J Vet Intern Med. 2021;35:834-842. 10.1111/jvim.16047 33621385 PMC7995432

[7] Wolters T, Roerink SH, Sterenborg R, et al. The effect of treatment on quality of life in patients with acromegaly: a prospective study. Eur J Endocrinol. 2020;182:319-331. 10.1530/EJE-19-0732 31958318

[8] Kyriakakis N, Lynch J, Gilbey SG, Webb SM, Murray RD. Impaired quality of life in patients with treated acromegaly despite long-term biochemically stable disease: results from a 5-years prospective study. Clin Endocrinol (Oxf). 2017;86:806-815. 10.1111/cen.13331 28316090

[9] Niessen S, Powney S, Guitian J, et al. Evaluation of a quality-of-life tool for cats with diabetes mellitus. J Vet Intern Med. 2010;24:1098-1105. 10.1111/j.1939-1676.2010.0579.x 20707839

[10] Scudder CJ, Hazuchova K, Gostelow R, et al. Pilot study assessing the use of cabergoline for the treatment of cats with hypersomatotropism and diabetes mellitus. J Feline Med Surg. 2021;23:131-137. 10.1177/1098612X20933213 32684121 PMC10741349

[11] Corsini A, Niessen SJ, Miceli DD, et al. Quality of life and response to treatment in cats with hypersomatotropism: the owners’ point of view. J Feline Med Surg. 2022; 1098612X221098718. 10.1177/1098612X221098718 PMC10812257 35616046

[12] Saunders B, Sim J, Kingstone T, et al. Saturation in qualitative research: exploring its conceptualization and operationalization. Qual Quant. 2018;52:1893-1907. 10.1007/s11135-017-0574-8 29937585 PMC5993836

[13] Taylor S, Caney S, Bessant C, Gunn-Moore D. Online survey of owners’ experiences of medicating their cats at home. J Feline Med Surg. 2022;24:1283-1293. 10.1177/1098612X221083752 35343808 PMC10812359

[14] Adamelli S, Marinelli L, Normando S, Bono G. Owner and cat features influence the quality of life of the cat. Appl Anim Behav Sci. 2005;94:89-98. 10.1016/j.applanim.2005.02.003

[15] Williams DL, Fred HM. Prevalence of feline cataract: results of a cross-sectional study of 2000 normal animals, 50 cats with diabetes and one hundred cats following dehydrational crises. Vet Ophthalmol. 2006;9:341-349. 10.1111/j.1463-5224.2006.00497.x 16939463

[16] Salgado D, Reusch C, Spiess B. Diabetic cataracts: different incidence between dogs and cats. Schweiz Arch Tierheilkund. 2000;142:349-353. https://pubmed.ncbi.nlm.nih.gov/10892302/10892302

[17] Guyonnet A, Donzel E, Bourguet A, Chahory S. Epidemiology and clinical presentation of feline cataracts in France: a retrospective study of 268 cases. Vet Ophthalmol. 2019;22:116-124. 10.1111/vop.12567 29508528

[18] Dimitri P . Treatment of acquired hypothalamic obesity: now and the future. Front Endocrinol. 2022;13:846880. 10.3389/fendo.2022.846880 PMC9019363 35464063

[19] Rose SR, Horne VE, Bingham N, Jenkins T, Black J, Inge T. Hypothalamic obesity: 4 years of the international registry of hypothalamic obesity disorders. Obesity. 2018;26:1727-1732. 10.1002/oby.22315 30296362 PMC6202209

[20] Aroch I, Mazaki-Tovi M, Shemesh O, et al. Central diabetes insipidus in five cats: clinical presentation, diagnosis and oral desmopressin therapy. J Feline Med Surg. 2005;7:333-339. 10.1016/j.jfms.2005.03.008 15927500 PMC10822417

[21] Bijsmans E, Jepson R, Syme H, et al. Psychometric validation of a general health quality of life tool for cats used to compare healthy cats and cats with chronic kidney disease. J Vet Intern Med. 2016;30:183-191. 10.1111/jvim.13656 26567089 PMC4913638

[22] Hadd MJ, Bienhoff SE, Little SE, et al. Safety and effectiveness of the sodium-glucose cotransporter inhibitor bexagliflozin in cats newly diagnosed with diabetes mellitus. J Vet Intern Med. 2023;37:915-924. 10.1111/jvim.16730 37148170 PMC10229323

[23] Cook AK, Behrend E. SGLT2 inhibitor use in the management of feline diabetes mellitus. J Vet Pharmacol Ther. 2025;48:19-30. 10.1111/jvp.13466 38954371 PMC11736986

