# Correction to: Owner points of view and perceived quality of life of diabetic cats pre- and post-hypophysectomy for hypersomatotropism

**DOI:** 10.1093/jvimsj/aalag060

**Published:** 2026-03-16

This is a correction to: Edward Shelton, Rosanne Jepson, David Church, Joe Fenn, Christopher Scudder, Owner points of view and perceived quality of life of diabetic cats pre- and post-hypophysectomy for hypersomatotropism, *Journal of Veterinary Internal Medicine*, Volume 40, Issue 1, January-February 2026, aalaf006, https://doi.org/10.1093/jvimsj/aalaf006

In the Abstract Results section, the last sentence should have a negative sign before the value 2.16. The sentence should read as follows: “The median AWIS before (−2.16; IQR, −4.23 to −1.17) and after (−1.27; IQR, −2.06 to −0.39) hypophysectomy were significantly less negative (*P* = .02)”. The value is correctly reported elsewhere in the article in Table 4 and the Results section.

Also, the Human ethics approval declaration is incorrect. The word “not” is missing and should read: “The authors declare human ethics approval was not required.”

We apologize for this error.

