# Supplementary material for: Owner points of view and perceived quality of life of diabetic cats pre- and post-hypophysectomy for hypersomatotropism
Source: J Vet Intern Med. 2026 Jan 21;40(1):aalaf006. doi: 10.1093/jvimsj/aalaf006 (PMC12881934; doi:10.1093/jvimsj/aalaf006)
Supplement: aalaf006_Supplementary_material_UPDATED [file aalaf006_supplementary_material_updated.docx]

Supplementary Table 1- Items included within adapted version of the DIAQoL Pet questionnaire.

| Item Number | Abbreviation | Item |
| --- | --- | --- |
| A | Alive status | Is your pet still alive? If your pet is sadly deceased, when did they pass away? |
| B | Breed | Cat breed |
| C | General QoL pet | In general, I feel that the quality of my pet’s life is: |
| D | Diabetes/ hypophysectomy dependent QoL pet | If your pet did not have diabetes and/or had hypophysectomy, his/her quality of life would be: |
| 1 | IWIS Worry | Do you worry about your pet's diabetes and/or medical conditions after hypophysectomy? |
| 2 | IWIS No treats | Do you ever feel you want to give your pet treats but don’t because of their diabetes and/or medical conditions after hypophysectomy? |
| 3 | IWIS conditions restrict life | Do you feel your life is restricted by your pet's diabetes and/or medical conditions after hypophysectomy? |
| 4 | IWIS medication discomfort | Does your pet ever react annoyed or in discomfort / pain when being administered medications (including insulin)? |
| 5 | IWIS medication worries | Do you ever worry about whether you have given the insulin / medications correctly? |
| 6 | IWIS resent medications | Do you resent having to give your pet insulin injections / medications? |
| 7 | IWIS Restrict your activities | Do you ever find the diabetes / management of your pet restricts or limits what you are doing or what you want to do, like going on holidays, away for weekends, away for the day/night, working? |
| 8 | IWIS More control | Do you ever feel you want to take more control of your pet's diabetes / condition on your own, without the help from vets and other people? |
| 9 | IWIS Pet’s moods | Do you think the diabetes and/or hormonal conditions after hypophysectomy affects your pet's moods? |
| 10 | IWIS Pet unwell | Does your pet ever feel unwell, tired or in any way negatively affected since insulin was started? |
| 11 | IWIS Boarding Kennels | Do you ever choose not to put your pet into boarding kennels because of the diabetes and/or because they had a hypophysectomy? |
| 12 | IWIS Friends and family | Do you ever choose not to leave your pet to stay with friends or family because of the diabetes and/or because they had a hypophysectomy? |
| 13 | IWIS Hypoglycemia | Does your pet every show signs of a low blood sugar (e.g. wobbliness, collapse)? |
| 14 | IWIS Active day | Do you ever choose not to take your pet with you on an active day (e.g. walking longer distances, going to the beach etc.) because of the diabetes? |
| 15 | IWIS Extra things (positive item) | Do you ever give your pet extra things, like snack, treats, extra attention or extra walks because of their diabetes and/or medical conditions after hypophysectomy? |
| 16 | IWIS Drinking | Does your pet still drink more than before their diagnosis (of diabetes)? |
| 17 | IWIS Urinate | Does your pet still urinate more than before the diagnosis? |
| 18 | IWIS Weight Loss | Is your pet still losing weight since treatment has begun (since they were diagnosed with diabetes)? |
| 19 | IWIS Future care | Do you ever feel worried you will not. be able to take care of your pet in the future because of the diabetes and /or conditions related to hypophysectomy? |
| 20 | IWIS Worry hypo | Do you ever feel worried about your pet suffering from an episode of low blood glucose? |
| 21 | IWIS Worry DKA | Do you ever feel worried about your pet suffering from an episode of ketoacidosis? |
| 22 | IWIS Worry vision | Do you ever worry about your pet getting vision problems due to cataracts or did you worry about this prior to your pet suffering from such problems? |
| 23 | IWIS Play less | Are you less inclined to play with your pet since their diabetes and/or since their hypophysectomy? |
| 24 | IWIS Play more (positive item) | Are you more inclined to play with your pet now that he/she has diabetes and/or since they had a hypophysectomy? |
| 25 | IWIS Social life | Do you ever find the need to fit your pet's diabetes and/or treatments needed after hypophysectomy into your social life?  e.g. carrying needles, food, medications, providing at a particular time |
| 26 | IWIS Working life | Do you ever find the need to fit your pet's diabetes and/or treatments needed after hypophysectomy into your working life? e.g. making special arrangements to work at specific times |
| 27 | IWIS Special Bond (positive item) | Do you feel you have a more special bond now with your pet now that you are managing his/her diabetes / medical conditions after hypophysectomy? |
| 28 | IWIS Costs | Do you ever worry about how much money your pet's diabetes and/or medical conditions after hypophysectomy costs you and your family? |
| E | DDAVP receive | Does your pet currently receive DDAVP/ desmopressin eye drops? |
| F | DDAVP worry | Do you worry more about administering DDAVP/ desmopressin eye drops than you do when administering insulin/ other medications required after hypophysectomy? |
| G | Appetite | Compared to before developing acromegaly or Cushing's, do you feel that your pet's appetite is currently: |
| H | Worry comorbidities | Do you worry about any concurrent health issues in your pet aside from diabetes and/or their care after hypophysectomy? |
| Free-text 1 | Concurrent health concerns | Please tell us more about any concurrent health concerns that you have with your pet if you wish to. |
| I | Proceed again | Given the choice again, would you proceed with hypophysectomy surgery again? |
| Free-text 2 | Additional comments | Is there any else you would like to say about your experiences of life with a pet with diabetes / experiences of life with a pet who has undergone hypophysectomy? |

Supplementary Table 2- Health concerns mentioned by 17 owners in response to the question ‘Please tell us more about any concurrent health concerns that you have with your pet if you wish to’.

| Health concern mentioned by owner | N (%)* |
| --- | --- |
| Cardiac disease | 2 (11.8) |
| Dental disease | 1 (5.9) |
| Dermatological disease | 1 (5.9) |
| Gastrointestinal disease | 2 (11.8) |
| Osteoarthritis and/or mobility concerns | 4 (23.5) |
| Increased appetite | 2 (11.8) |
| Renal disease | 1 (5.9) |
| Weight gain | 5 (29.4) |
| Hepatobiliary disease and/or pancreatitis | 2 (11.8) |
| Upper respiratory tract infections | 1 (5.9) |
| Neoplasia | 1 (5.9) |

* N reflects number of respondents mentioning the specific health concern and percentage reflects the percent of respondents raising this concern.

Supplementary Table 3- Summary of answers of 27 respondents in retrospective cohort to the free-text question ‘Is there anything else you would like to say about your experiences of life with a pet with diabetes / experiences of life with a pet who has undergone hypophysectomy?’

| Point raised | N (%) |
| --- | --- |
| Without hypophysectomy the owner felt they either wouldn’t have their cat, would have euthanised their cat or that hypophysectomy had saved their cat’s life | 4 (14.8) |
| Owner worried that their cat was not as active and sleeps more | 1 (3.7) |
| Owner felt that hypophysectomy had extended their cat’s life | 2 (7.4) |
| Owner described hypophysectomy as life changing for their cat | 1 (3.7) |
| Owner commented that even with medications required, hypophysectomy was the right decision | 1 (3.7) |
| Owner commented that their cat was needle phobic and the medications required post hypophysectomy were no issue | 1 (3.7) |
| Owner commented that their cat was not the same cat as before hypersomatotropism, however they were pleased they went ahead with hypophysectomy and felt their cat had a good quality of life | 1 (3.7) |
| Owner highlighted excessive weight gain in their cat leading to reduced mobility | 2 (7.4) |
| Owner described their cat having a ravenous appetite that they had expected to resolve post treatment and that this had been difficult to manage | 1 (3.7) |
| Owner referenced lack of knowledge from primary care vets on their cat’s condition | 1 (3.7) |
| Owner referenced difficulty in administering desmopressin eye drops and an improvement in ease of administration after switching to oral tablets | 3 (11.1) |
| Owner mentioned the commitment required to daily medication routine and either finding a cattery to administer the medication or boarding cat with primary care practice when going away | 2 (7.4) |
| Owner mentioned impact of medications on social life and going away on holiday | 2 (7.4) |
| Owner mentioned anxiety around leaving their cat with friends or in a cattery | 1 (3.7) |
| Owner mentioned things were easier since their cat no longer required desmopressin | 1 (3.7) |
| Owner mentioned concern immediately post-operatively that their cat could develop an Addisonian crisis | 1 (3.7) |
| Owner commented that being able to use a continuous glucose monitor (FreeStyle Libre) at home gave them peace of mind | 1 (3.7) |
| Owner commented that they were relieved their cat went into diabetic remission as they had not felt confident with home glucose monitoring | 1 (3.7) |

Supplementary Table 4- Health concerns mentioned by 7 owners pre-hypophysectomy and 6 owners post-hypophysectomy in response to the question ‘Please tell us more about any concurrent health concerns that you have with your pet if you wish to’.

| Health concern mentioned by owner | N (%)* |
| --- | --- |
| Pre-hypophysectomy |  |
| Cardiac disease | 3 (42.9) |
| Increased thirst | 1 (14.3) |
| Weight loss | 1 (14.3) |
| Gastrointestinal disease | 1 (14.3) |
| Osteoarthritis and/or mobility concerns including neuropathy | 3 (42.9) |
| Increased appetite | 2 (28.6) |
| Renal disease | 1 (14.3) |
| Urinary tract disease | 2 (28.6) |
| Ocular disease | 3 (42.9) |
| Upper respiratory tract infections | 1 (14.3) |
| Other endocrine disease | 1 (14.3) |
| Neoplasia | 1 (14.3) |
| Post-hypophysectomy |  |
| Overgrooming (present since earlier in life) | 1 (16.7) |
| Renal disease | 1 (16.7) |
| Weight gain | 1 (16.7) |
| Abdominal distension | 1 (16.7) |
| Possible lower jaw overgrowth affecting eating | 1 (16.7) |
| Post-operative infection | 1 (16.7) |

* N reflects number of respondents mentioning the specific health concern and percentage reflects the percent of respondents raising this concern.

Supplementary Table 5- Summary of answers of 7 respondents pre-hypophysectomy and 9 respondents post-hypophysectomy to the free-text question ‘Is there anything else you would like to say about your experiences of life with a pet with diabetes / experiences of life with a pet who has undergone hypophysectomy?’

| Point raised | N (%) |
| --- | --- |
| Pre-hypophysectomy |  |
| Owner mentioned excessive hunger and/or hunger aggression affecting their relationship with their cat | 2 (28.6) |
| Owner mentioned difficulty in seeing no response from their cat to insulin injections | 3 (42.9) |
| Owner commented on feeling helpless around the time taken to reach a diagnosis and then wait for hypophysectomy surgery and mentioned feeling even worse for owners who can’t afford hypophysectomy | 1 (14.3) |
| Owner mentioned that achievement of diabetic remission would be their hope post-hypophysectomy | 1 (14.3) |
| Owner mentioned that things had been tough since their cat was diagnosed with diabetes and they were hoping for improved quality of life for their cat | 1 (14.3) |
| Post-hypophysectomy |  |
| Owner commented that they were delighted with their cat’s progress | 1 (11.1) |
| Owner commented that dealing with medications after hypophysectomy was a breeze compared to treating diabetes with insulin injections | 1 (11.1) |
| Owner commented that they found medication administration overwhelming initially but it became less stressful with time | 2 (22.2) |
| Owner mentioned the demand of medications on their life, however said they would absolutely go ahead with hypophysectomy again as their cat was much happier post-surgery | 1 (11.1) |
| Owner commented that living with a cat who had undergone hypophysectomy was relatively easy due to an excellent relationship with their local vet | 1 (11.1) |
| Owner commented that owners need to be aware of the time allocated and long-term commitment to pre-operative assessments, hospitalisation and post-operative checks, however hypophysectomy was 100 percent worth it and their cat would probably be dead without it | 1 (11.1) |
| Owner was delighted by how their cat’s quality of life had improved but mentioned their cat had an infection post-operatively which was extremely worrying for them | 1 (11.1) |
| Owner commented that their cat’s recovery had been slower than they had expected but they had seen improvements and were less concerned about their cat’s health and wellbeing | 1 (11.1) |
| Owner commented that their cat was extremely polyuric and polydipsic which was difficult for them and that desmopressin eye drop administration to their cat could be challenging | 1 (11.1) |

Supplementary material- Telephone interview script

‘’ *Good morning/ afternoon (owners name), I’m a vet calling from the internal medicine team at the Royal Veterinary College. I’m currently undertaking a research project looking at quality of life in cats after hypophysectomy surgery and I was to discuss (pet’s name) quality of life because they had hypophysectomy surgery with us in (month and year of surgery). There is no obligation to participate and you can withdrawal consent at any time. Do you consent to participate?’’*

- If owner declines speaking then the call will be cut short in a polite and thoughtful manner and a note made not to contact the owner by email with the study surveys.
- If owner is happy to talk then the owner will be thanked for agreeing to talk and it will be checked that the owner is happy to speak now: *‘’ Thank you for agreeing to speak, it is much appreciated. We’ll be sending some surveys out by email in the coming months, but also felt it would be beneficial to speak with a selection of owners in person to help us ensure that our survey highlights the information that is most important to owners. I’ll ask you a few questions about (pet’s name) and am very interested to hear your thoughts. Is now an ok time for us to chat for 20-30 minutes or so, or would it be better to call back at another time?’’*
- If owner not free to speak at that time then a convenient time to call the owner back will be arranged. If owner happy to proceed with telephone interview at that time, they will be thanked and the conversation will continue: *‘’ Just to make you aware, our phone lines are recorded but recordings will not be routinely accessed or shared within or outside of the RVC. I’ll make some notes of what we discuss to help us with this research project, but all information will be anonymised. ‘’*
- Once owner has consented to proceed, the interview will begin with the open question: *‘’ Please could you tell me a bit about how you feel about hypophysectomy surgery after (pet’s name) experience?’’*
- The interview will follow in a fluid manner with follow up discussion dictated by the owners response, specific questions asked may include:

*‘’ How do you feel about (pet’s name)’s quality of life now when compared to before the hypophysectomy surgery?’’*

*‘’How do you feel about giving ongoing medications after hypophysectomy in comparison to insulin injections?’’*

*‘’ Do you worry at all about (pet’s name) after his/her hypophysectomy?’’*

*‘’ Are there any differences for you in (pet’s name) behaviour after his/ her hypophysectomy?’’ ‘’Do you feel like these changes are positive or negative for you and (pet’s name)?’’*

*‘’If you could make the decision again would you still go ahead with hypophysectomy surgery?’’*

- Empathetic and understanding responses will be given to the owner’s answers but commenting on further details (for example if a cat has not responded to treatment or has suffered from unrelated illness) will be avoided.
- In the unlikely event that an owner is contacted by phone when their cat has recently passed away, empathy and appropriate responses to owner questions will be offered. Empathetic language and avoidance of the terms ‘died’, ‘dead’ and ‘deceased’ will be avoided with the term ‘passed away’ favoured. If the owner appears extremely upset (not an expected scenario) then they will be directed to a pet bereavement support line .

Supplementary material – Questionnaire

**Quality of life assessment for pets who have / had diabetes mellitus and hypophysectomy**

***Thank you for undertaking this questionnaire. This survey takes around 15 minutes to complete. The survey enquires about your pet's quality of life following hypophysectomy, which some may find upsetting if your pet has passed away. If your pet has passed and you do not wish to complete the survey then please close this window. Please only complete this survey is you cat has undergone hypophysectomy or is due to undergo hypophysectomy in the coming weeks.*

*The aim of this survey is to achieve a better understanding of the advantages and disadvantages of performing hypophysectomy to treat cats with pituitary disease. We hope to be able to use this data to influence our clinical recommendations and may publish the results at a veterinary conference / in a veterinary journal. 

The data collected will be anonymised, and stored on the secure servers of the Royal Veterinary College for no longer than five years. Only Royal Veterinary College staff and students involved in this research will have access to the data. Your participation is voluntary and consent to be included can be withdrawn at any time.*
 *Thank you for undertaking this survey. We hope the data will help us to understand how we may can improve the quality of life of diabetic cats with pituitary disease. If you have any questions please contact us.***

**This section will be to record you pet's details**

1.What is your pet's name (please also include your surname)?

2.If known, please record your pet's Royal Veterinary College hospital number

3. Is your pet still alive?

Yes/ No

4.If your pet is sadly deceased, when did they pass away?

5.Is your pet a cat or dog?

6.Cat breed (if applicable)

Select your answer

7. Dog breed (if applicable)

Select your answer

**This section will focus on your pet's quality of life**

8. In general: I feel that the quality of my pet's life is:
*(please select the preferred option) As good as it could possibly be/ Good/Fairly good/ Neither good nor bad/ Fairly poor/ Poor/ As poor as it could possibly be/ Don't know*

9. If your pet did not have diabetes and/or had hypophysectomy, his/her quality of life would be:

Select your answer: A great deal better/ Quite a lot better/ A little better/ The same/ A little worse/ A great deal worse/ Don't know

**Please answer the following questions about your pet**

10.Do you worry about your pet's diabetes and/or medical conditions after hypophysectomy?

A lot/ Quite a lot/ A little/ Not at all/ Don't know/ My pet is not diabetic

*For the purposes of assigning frequency scores to this question: A lot= all the time, quite a lot= often, a little = occasionally, not at all/ don’t know/ my pet is not diabetic = never/ don’t know

11.For me and my pet, the effect of this worrying is:

Very important/ Important/ Moderately important/ Low importance/ Not at all important/ Not applicable

12.Do you ever feel you want to give your pet treats but don’t because of their diabetes and/or medical conditions after hypophysectomy?

All the time/ Often/ Occasionally/ Never/ Don't know

13.For me and my pet, this issue over feeding treats is:

Very important/ Important/ Moderately important/ Low importance/ Not at all important/ Not applicable

14.Do you feel your life is restricted by your pet's diabetes and/or medical conditions after hypophysectomy?

All the time/ Often/ Occasionally/ Never/ Don't know

15.For me, this issue is:

Very important/ Important/ Moderately important/Low importance/ Not at all important/ Not applicable

16.Does your pet ever react annoyed or in discomfort / pain when being administered medications (including insulin)?

All the time/ Often/ Occasionally/ Never/ Don't know

17.For me and my pet, the problem of administering medications is:

Very important/ Important/ Moderately important/ Low importance/ Not at all important/ Not applicable

18.Do you ever worry about whether you have given the insulin / medications correctly?

All the time/ Often/ Occasionally/ Never/ Don't know/ Not applicable

19.For me, the issue of worry about correct insulin / medication administration is:

Very important/ Important/ Moderately important/ Low importance/ Not at all important/ Not applicable

20.Do you resent having to give your pet insulin injections / medications?

All the time/ Often/ Occasionally/ Never/ Don't know

21.For me, this issue is:

Very important/ Important/ Moderately important/ Low importance/ Not at all important/ Not applicable

22.Do you ever find the diabetes / management of your pet restricts or limits what you are doing or what you want to do, like going on holidays, away for weekends, away for the day/night, working?

All the time/ Often/ Occasionally/ Never/ Don't know

23.For me, this issue is:

Very important/ Important/ Moderately important/ Low importance/ Not at all important/ Not applicable

24.Do you ever fell you want to take more control of your pet's diabetes / condition on your own, without the help from vets and other people?

All the time/ Often/ Occasionally/ Never/ Don't know

25.For me, this issue is:

Very important/ Important/ Moderately important/ Low importance/ Not at all important/ Not applicable

26.Do you think the diabetes and/or hormonal conditions after hypophysectomy affects your pet's moods?

All the time/ Often/ Occasionally/ Never/ Don't know

27.For me, this issue is:

Very important/ Important/ Moderately important/ Low importance/ Not at all important/ Not applicable

28.Does your pet ever feel unwell, tired or in any way negatively affected since insulin was started?

All the time/ Often/ Occasionally/ Never/ Don't know

29.For me, this issue is:

Very important/ Important/ Moderately important/ Low importance/ Not at all important/ Not applicable

30.Do you ever choose not to put your pet into boarding kennels because of the diabetes and/or because they had a hypophysectomy?

All the time/ Often/ Occasionally/ Never/ Don't know

31.For me, this issue is:

Very important/ Important/ Moderately important/ Low importance/ Not at all important/ Not applicable

32.Do you ever choose not to leave your pet to stay with friends or family because of the diabetes and/or because they had a hypophysectomy?

All the time/ Often/ Occasionally/ Never/ Don't know

33.For me, this issue is:

Very important/ Important/ Moderately important/ Low importance/ Not at all important/ Not applicable

34.Does your pet every show signs of a low blood sugar (e.g. wobbliness, collapse)?

All the time/ Often/ Occasionally/ Never/ Don't know

35.For me, this issue is:

Very important/ Important/ Moderately important/ Low importance/ Not at all important/ Not applicable

36.Do you ever choose not to take your pet with you on an active day (e.g. walking longer distances, going to the beach etc.) because of the diabetes?

All the time/ Often/ Occasionally/ Never/ Don't know

37.For me, this issue is:

Very important/ Important/ Moderately important/ Low importance/ Not at all important/ Not applicable

38.Do you ever give your pet extra things, like snack, treats, extra attention or extra walks because of their diabetes and/or medical conditions after hypophysectomy?

All the time/ Often/ Occasionally/ Never/ Don't know

39.For me, this issue is:

Very important/ Important/ Moderately important/ Low importance/ Not at all important/ Not applicable

40.Does your pet still drink more than before their diagnosis (of diabetes)?

All the time/ Often/ Occasionally/ Never/ Don't know

41.For me, this issue is:

Very important/ Important/ Moderately important/ Low importance/ Not at all important/ Not applicable

42.Does your pet still urinate more than before the diagnosis?

All the time/ Often/ Occasionally/ Never/ Don't know

43.For me, this issue is:

Very important/ Important/ Moderately important/ Low importance/ Not at all important/ Not applicable

44.Is your pet still losing weight since treatment has begun (since they were diagnosed with diabetes)?

All the time/ Often/ Occasionally/ Never/ Don't know

45.For me, this issue is:

Very important/ Important/ Moderately important/ Low importance/ Not at all important/ Not applicable

46.Do you ever feel worried you will not. be able to take care of your pet in the future because of the diabetes and /or conditions related to hypophysectomy?

All the time/ Often/ Occasionally/ Never/ Don't know

47.For me, this issue is:

Very important/ Important/ Moderately important/ Low importance/ Not at all important/ Not applicable

48.Do you ever feel worried about your pet suffering from an episode of low blood glucose?

All the time/ Often/ Occasionally/ Never/ Don't know

49.For me, this issue is:

Very important/ Important/ Moderately important/ Low importance/ Not at all important/ Not applicable

50.Do you ever feel worried about your pet suffering from an episode of ketoacidosis?

All the time/ Often/ Occasionally/ Never/ Don't know

51.For me, this issue is:

Very important/ Important/ Moderately important/ Low importance/ Not at all important/ Not applicable

52.Do you ever worry about your pet getting vision problems due to cataracts or did you worry about this prior to your pet suffering from such problems?

All the time/ Often/ Occasionally/ Never/ Don't know

53.For me, this issue is:

Very important/ Important/ Moderately important/ Low importance/ Not at all important/ Not applicable

54.Are you **less** inclined to play with your pet since their diabetes and/or since their hypophysectomy?

All the time/ Often/ Occasionally/ Never/ Don't know

55.For me, this issue is:

Very important/ Important/ Moderately important/ Low importance/ Not at all important/ Not applicable

56.Are you **more** inclined to play with your pet now that he/she has diabetes and/or since they had a hypophysectomy?

All the time/ Often/ Occasionally/ Never/ Don't know

57.For me, this issue is:

Very important/ Important/ Moderately important/ Low importance/ Not at all important/ Not applicable

58.Do you ever find the need to fit your pet's diabetes and/or treatments needed after hypophysectomy into your social life?
*e.g. carrying needles, food, medications, providing at a particular time*

All the time/ Often/ Occasionally/ Never/ Don't know

59.For me, this issue is:

Very important/ Important/ Moderately important/ Low importance/ Not at all important/ Not applicable

60.Do you ever find the need to fit your pet's diabetes and/or treatments needed after hypophysectomy into your working life?
*e.g. making special arrangements to work at specific times*

All the time/ Often/ Occasionally/ Never/ Don't know

61.For me, this issue is:

Very important/ Important/ Moderately important/ Low importance/ Not at all important/ Not applicable

62.Do you feel you have a more special bond now with your pet now that you are managing his/her diabetes / medical conditions after hypophysectomy?

All the time/ Often/ Occasionally/ Never/ Don't know

63.For me, this issue is:

Very important/ Important/ Moderately important/ Low importance/ Not at all important/ Not applicable

64.Do you ever worry about much money your pet's diabetes and/or medical conditions after hypophysectomy costs you and your family?

All the time/ Often/ Occasionally/ Never/ Don't know

65.For me and my family, this issue is:

Very important/ Important/ Moderately important/ Low importance/ Not at all important/ Not applicable

**Please use this section to tell us about your experiences**

66.Does your pet currently receive DDAVP/ desmopressin eye drops?

Yes/ No

67.Do you worry more about administering DDAVP/ desmopressin eye drops than you do when administering insulin/ other medications required after hypophysectomy?

All the time / Often/ Occasionally / Never/ Don't know / Not applicable

68.Compared to before developing acromegaly or Cushing's,  do you feel that your pet's appetite is currently:

Very decreased/ Slightly decreased/ Normal/ Slightly increased / Very increased

69.Do you worry about any concurrent health issues in your pet aside from diabetes and/or their care after hypophysectomy?

Yes / No

70.Please tell us more about any concurrent health concerns that you have with your pet if you wish to.

71.Given the choice again, would you proceed with hypophysectomy surgery again?

Yes/ No/ Unsure/ My pet hasn't had hypophysectomy surgery yet

72.Is there anything else you would like to say about your experiences of life with a pet with diabetes / experiences of life with a pet who has undergone hypophysectomy?

73.Please give any feedback on this questionnaire in this section
